# Human peritoneal tight junction, transporter and channel expression in health and kidney failure, and associated solute transport

**DOI:** 10.1038/s41598-023-44466-z

**Published:** 2023-10-13

**Authors:** Eszter Levai, Iva Marinovic, Maria Bartosova, Conghui Zhang, Betti Schaefer, Hanna Jenei, Zhiwei Du, Dorota Drozdz, Günter Klaus, Klaus Arbeiter, Philipp Romero, Vedat Schwenger, Constantin Schwab, Attila J. Szabo, Sotirios G. Zarogiannis, Claus Peter Schmitt

**Affiliations:** 1 Division of Pediatric Nephrology, Center for Pediatric and Adolescent Medicine, University Hospital Heidelberg, Im Neuenheimer Feld 430, 69120 Heidelberg, Germany; 2 Pediatric Center, MTA Center of Excellence, Semmelweis University, Budapest, Hungary; 3 HUNREN SE Pediatrics and Nephrology Research Group, Budapest, Hungary; 4 Jagiellonian University Medical College, Krakow, Poland; 5 KfH Pediatric Kidney Center, Marburg, Germany; 6 Department of Pediatrics and Adolescent Medicine, Medical University Vienna, Vienna, Austria; 7 Division of Pediatric Surgery, Department of General, Visceral and Transplantation Surgery, University Hospital Heidelberg, Heidelberg, Germany; 8 Department of Nephrology, Klinikum der Landeshauptstadt Stuttgart, Stuttgart, Germany; 9 Institute of Pathology, Heidelberg University, Heidelberg, Germany; 10 Department of Physiology, Faculty of Medicine, University of Thessaly, Larissa, Greece

**Keywords:** Translational research, Peritoneal dialysis, Chronic inflammation, Paediatric research, Molecular medicine

## Abstract

Next to the skin, the peritoneum is the largest human organ, essentially involved in abdominal health and disease states, but information on peritoneal paracellular tight junctions and transcellular channels and transporters relative to peritoneal transmembrane transport is scant. We studied their peritoneal localization and quantity by immunohistochemistry and confocal microscopy in health, in chronic kidney disease (CKD) and on peritoneal dialysis (PD), with the latter allowing for functional characterizations, in a total of 93 individuals (0–75 years). Claudin-1 to -5, and -15, zonula occludens-1, occludin and tricellulin, SGLT1, PiT1/SLC20A1 and ENaC were consistently detected in mesothelial and arteriolar endothelial cells, with age dependent differences for mesothelial claudin-1 and arteriolar claudin-2/3. In CKD mesothelial claudin-1 and arteriolar claudin-2 and -3 were more abundant. Peritonea from PD patients exhibited increased mesothelial and arteriolar claudin-1 and mesothelial claudin-2 abundance and reduced mesothelial and arteriolar claudin-3 and arteriolar ENaC. Transperitoneal creatinine and glucose transport correlated with pore forming arteriolar claudin-2 and mesothelial claudin-4/-15, and creatinine transport with mesothelial sodium/phosphate cotransporter PiT1/SLC20A1. In multivariable analysis, claudin-2 independently predicted the peritoneal transport rates. In conclusion, tight junction, transcellular transporter and channel proteins are consistently expressed in peritoneal mesothelial and endothelial cells with minor variations across age groups, specific modifications by CKD and PD and distinct associations with transperitoneal creatinine and glucose transport rates. The latter deserve experimental studies to demonstrate mechanistic links.

Clinical Trial registration: The study was performed according to the Declaration of Helsinki and is registered at www.clinicaltrials.gov (NCT01893710).

## Introduction

The peritoneum is a thin serosal membrane lining the abdominal cavity and organs with a surface area similar to body surface area^1^. It consists of the mesothelial cell monolayer lining the basal membrane together with the fibrous submesothelium, which contains blood and lymphatic vessels and nerves. The peritoneum provides nutrition and mechanical support to abdominal organs, protects from frictions and adhesions and regulates local homeostasis including inflammatory, fibrotic and angiogenic processes and exchanges abdominal fluids^2^. It is involved in internal organ development through biochemical cues that drive and sustain cell transition^3,4^ and regulates pathophysiological processes such as peritoneal tumor progression and post-infectious and post-interventional adhesions^5^. Mesothelial cells induce fibrosis in cases of sustained noxious stimuli by formation of cell protrusions that adjoin the opposing mesothelial surfaces (visceral and parietal peritoneal surfaces)^6,7^. Malignant mesothelial cells form protrusions in the context of extracellular matrix cues^8^. In all these conditions mesothelial cells rearrange their cell-to-cell communication involving tight junctions (TJ)^9–11^.

For more than half a century the human peritoneum has been used as an endogenous, semipermeable dialysis membrane for patients with end stage kidney disease. As compared to hemodialysis, peritoneal dialysis (PD) has important advantages as a renal replacement therapy since it does not require a permanent vascular access, and it is a home-based therapy with superior compatibility with professional and social life. PD is the preferred dialysis modality in children and is increasingly applied in adults^12,13^ but has major limitations regarding toxin-, salt- and water removal, and progressive deterioration of the peritoneal membrane integrity that limits its sustainability^14,15^. The molecular counterparts of the dialytic transperitoneal transport are largely unknown.

Transmembrane transport across epithelial and endothelial barriers involves the paracellular and transcellular route. The paracellular transport pathway is established by permselective TJs that are mainly constituted by claudins (CLDNs) along with intracellular accessory proteins^16^. Permeability is controlled by claudins and the TJ-associated MARVEL proteins occludin (OCL), tricellulin (TriC) and marvelID3. Claudin 2, -4 and 15 are pore forming and facilitate the paracellular passage of water and sodium (-2 and -15) and chloride (-4), while claudin-1, -3, -5 have sealing functions thus reducing the paracellular conductance^17^. Zonula occludens (ZO) proteins connect TJ to the actin cytoskeleton. Tricellulin seals the barrier at the conjunction of three cells hindering the passage of macromolecules. The function of occludin is partially understood; occludin knock-out in mice did not alter intestinal barrier function, but regulates paracellular permeability under hydrostatic pressure changes^16,18^.

The transcellular pathway involves plasma membrane water and ion channels and transporters such as water channel aquaporin-1 (AQP1), the epithelial sodium channel (ENaC), the sodium-glucose co-transporters-1 and -2 (SGLT1 and SGLT2) and sodium/phosphate cotransporter PiT1/SLC20A1 (PiT1). Albeit the great functional impact of tight junction, transcellular transporter and channel proteins is well known, in the peritoneum only peritoneal AQP1 has systematically been studied to date in vivo. AQP1 is age-independently expressed in the mesothelial cell layer and the peritoneal capillaries and arterioles^19^. In mice with global AQP1-knock-out, peritoneal water transport is reduced by 50%^20–22^. AQP1 promoter variants influence ultrafiltration and are associated with technique failure and mortality rates in patients on chronic PD^23^. Detached effluent mesothelial cells cultured in vitro express CLDN-1, -2, -8, occludin, and ZO-1 at lower levels when isolated from patients with high versus low transporter status^24^. TJ proteins can also be quantified in dialysate effluent and may reflect the peritoneal small solute transport, albeit it is unclear in how fat these proteins also reflect the PD fluid induced insult to the mesothelium and originate from detaching mesothelial cells^25^.Glucose and oxidative stress, but not the glucose polymer icodextrin, reduce ZO-1, occludin, and claudin-1 in cultured human primary peritoneal mesothelial cells^26,27^. Glucose substitutes xylitol and l-carnitine preserve ZO-1 membrane abundance in immortalized human mesothelial cells^28^. These findings illustrate the potential relevance of paracellular and the transcellular pathways in the peritoneum and justify their in-depth investigation in peritoneal tissue. We therefore quantified peritoneal TJ and transcellular ion channel and transporter proteins across age groups, and in children devoid of life-style and aging related bias, the regulation by chronic kidney disease (CKD) and PD, and the association with peritoneal transport function.

## Results

### Localization and quantification of peritoneal TJ in the healthy peritoneum

**Figure 1:**
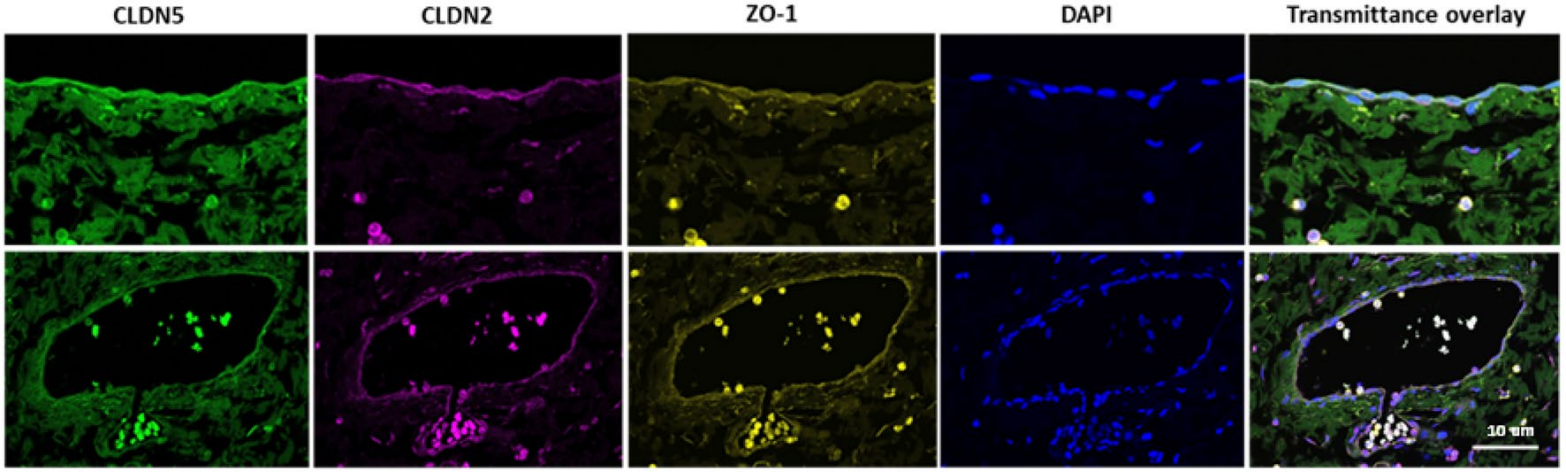
CLDN5, CLDN2 and ZO-1 co-expression in the parietal peritoneum. Peritoneal mesothelium (upper row) and arteriolar endothelium (lower row) are shown. ZO-1 and claudin-2 proteins were also expressed in blood leucocytes.

Peritoneal tissues of 46 patients with normal renal function, aged 0–75 years underwent immunohistochemical staining and digital quantification of the TJ proteins claudin-1, -3, -5 with sealing function, claudin-2, -4, -15 with pore forming function, ZO-1 connecting the TJ to the actin cytoskeleton, OCL, an important regulatory protein and TriC, prevalent in the junction of three cells. Transcellular channel and transporter proteins quantitated were ENaC (sodium channel), PiT1 (sodium-phosphate co-transporter), and SGLT1 (sodium-glucose transporter). All these proteins were consistently detected in mesothelial and arteriolar endothelial cells (Fig. 1, Table S1) with age dependent differences in abundance for mesothelial claudin-4 and arteriolar claudin-1 and -2 (Table S2), but without following a linear age correlation. Since children are largely devoid of aging and life-style related factors and allow for more sensitive and specific studies, subsequent studies were limited to pediatric patients.

### Peritoneal morphology, TJ and transcellular ion transporting proteins and channels in CKD5 and PD

Biochemical parameters were driven by kidney function, with no differences between children with CKD5 and on PD (Table 1). Digital histomorphometry demonstrated some inflammatory cell infiltration of the peritoneum and activation of fibroblast and profibrotic activity in children with CKD5 and vascular lumen narrowing. In patients on PD peritoneal mesothelial cell coverage was reduced, mesothelial-mesenchymal transitioned cells were present in the submesothelium, which was more vascularized, and was infiltrated by inflammatory cells, i.e. macrophages and leukocytes (Table 2, Figure S1B). TJ and transcellular ion channels and transporter proteins are expressed by immune cells ^29^ and we reconfirmed this finding in blood leukocytes for the pore-forming claudin-2 (Figure S1A). To exclude an analytic bias by the degree of peritoneal inflammatory cell infiltration, we limited TJ and cellular transporter studies to the mesothelial monolayer and the arterioles.

**Table 1:** Pediatric patient characteristics and biochemical parameters in individuals with normal kidney function, CKD5 and on PD.

	Controls (n = 23)	CKD 5 (n = 23)	PD (n = 24)	*p*-value*
Age (years)	5.7 (1.5, 10.8)	8.2 (2.1, 14.8)	8.6 (2.4, 13.2)	0.41
Gender (% female)	48	26	42	0.19
Body surface area (m^2^)	0.8 (0.5, 1.2)	0.8 (0.5, 1.4)	0.9 (0.5, 1.3)	0.92
Hemoglobin (g/dL)	12.8 (12.0, 13.4)	11 (9.2, 12.5)^aa^	10.6 (9.8, 11.8)^aa^	**0.0001**
Serum calcium (mmol/L)	2.4 (2.3, 2.6)	2.4 (2.1, 2.6)	2.4 (2.2, 2.5)	0.7
Serum phosphate (mmol/L)	1.6 (1.1, 1.9)	1.7 (1.5, 2.0)	1.7 (1.4, 2.0)	0.53
Serum albumin (g/L)	44.8 (44.8, 45.0)	39.5 (29.4, 41.5)^a^	34.5 (31.3, 38.3)^aa^	**0.004**
Serum creatinine (mg/dL)	0.3 (0.2, 0.5)	5.7 (3.7, 7.0)^aaa^	7.0 (4.8, 11.1)^aaa^	** < 0.0001**
Blood urea nitrogen (mg/dL)	n.d	57 (37, 76)	56 (41, 64)	n/a

* ANOVA/Kruskal–Wallis test as appropriate, followed by post hoc group comparisons corrected for multiple testing. Data is expressed as median and interquartile range. Superscript “a” indicates a significant difference to control group, *p* < 0.05, “aa” *p* < 0.01, “aaa” *p* < 0.001. There were no significant differences relative to the CKD5 group.

**Table 2:** Digital histomorphometry of the parietal peritoneum of children with normal kidney function, with CKD5 and children on PD.

	Controls (n = 23)	CKD 5 (n = 23)	PD (n = 24)	*p*-value*	CKD5 vs PD^+^
Mesothel coverage (0–6)	5.0 (3.5, 6.0)	5.0 (5.0, 6.0)	3.0 (1.5, 6.0)	**0.04**	0.06
Submesothelial thickness (µm)	250 (150, 400)	325 (213, 395)	307 (265, 503)	0.07	0.30
Submesothelial microvessel density (/mm^2^)	80 (56.5, 185)	126 (80, 150)	161 (111,227)^a^	**0.02**	0.24
Lymphatic vessel density (/mm^2^)	37.4 (19.9, 54.3)	26.1 (19, 48.2)	22.5, (8.7, 45.9)	0.26	1.0
Diffuse podoplanin staining (% patients)	0	0	21	0.08	0.97
Blood microvessel density (/mm^2^)	61 (38, 160)	98 (64, 117)	153 (64, 219)^a^	**0.01**	0.22
Total endothelial surface area (µm^2^/um^3^)	3.5 (2.1, 4.7)	5.7 (4.3, 7.4)	9.6 (6.1, 13.7)^aaa^	** < 0.0001**	**0.04**
Lymphatic endothelial surface area (µm^2^/um^3^)	1.4 (1.1, 3.4)	2.2 (1.5, 2.8)	2.4 (1.4, 4.2)	0.59	1.0
Blood microvessel endothelial surface area (µm^2^/ µm^3^)	1.6 (1.1, 3.4)	3.4 (2.4, 4.5)	7.2 (5.7, 10.8)^aaa^	**0.0009**	0.05
L/V ratio	0.61 (0.56, 0.77)	0.53 (0.47, 0.66)^**a**^	0.46 (0.37, 0.61)^aaa^	** < 0.0001**	0.25
ASMA positive cells (% patients)	0	26.1	45.8^aaa^	**0.002**	0.28
ASMA score (0–3)	0 (0, 0)	0 (0, 1)	1.0 (0, 2)^aaa^	**0.0004**	0.08
CD45 positive cells (% patients)	0	13	71^aaa^	** < 0.0001**	** < 0.0001**
CD45 score (0–3)	0 (0, 0)	0 (0, 1)	1 (0, 2)^aaa^	** < 0.0001**	** < 0.0001**
CD68 positive cells (% patients)	0	8.7	58.3^aaa^	** < 0.0001**	**0.0001**
CD68 score (0–3)	0 (0, 0)	0 (0, 1)	1 (0, 2)^aaa^	** < 0.0001**	** < 0.0001**
Fibrin deposits (% patients)	0	4.3	16.7	0.07	0.28
EMT positive (% patients)^#^	0	0	42^aaa^	** < 0.0001**	**0.0003**
EMT (cells/mm^2^)^#^	n.a	n.a	25 (18, 53)	**n.a**	**n.a**

L/V ratio = lumen diameter/vessel ratio, ASMA = alpha smooth muscle actin, EMT = epithelial to mesenchymal transition. ^#^ only EMT positive patients included. n.a. = not applicable. * ANOVA/Kruskal–Wallis test as appropriate. + T-test or Mann–Whitney test as appropriate. Data is expressed as median and interquartile range. Superscript “a” indicates a significant difference to control group. “a” indicates *p* < 0.05, “aa” *p* < 0.01, “aaa” *p* < 0.001.

**Figure 2:**
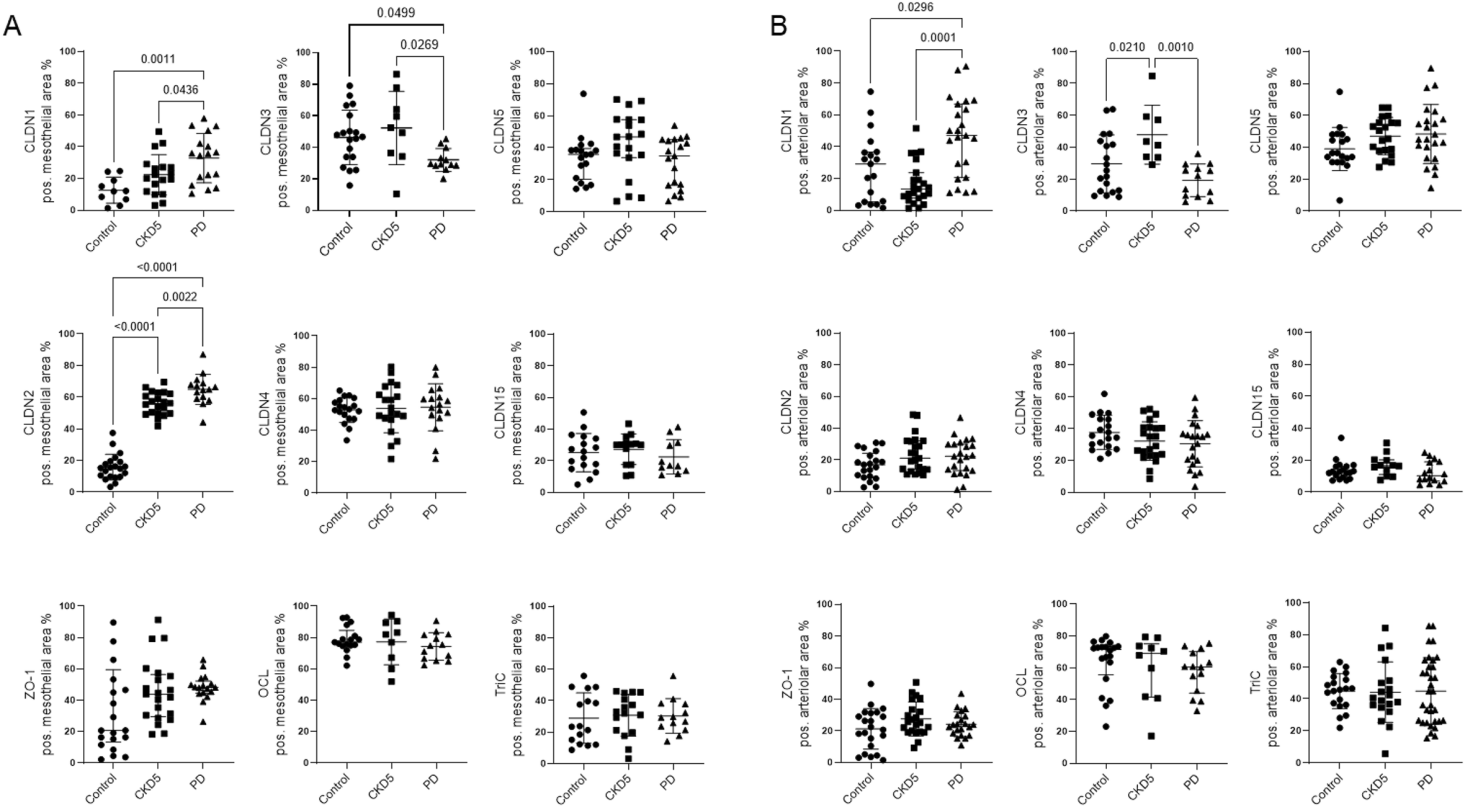
Tight junction protein abundance in children with normal kidney function, children with chronic kidney disease (CKD5) and on peritoneal dialysis (PD). Abundance of sealing claudins (CLDN1, CLDN3, CLND5), pore-forming claudins (CLDN2, CLDN4, CLDN15) and ZO-1, OCL and TriC in the mesothelial (**A**) and in the arteriolar area (**B**). CKD5 patients showed a higher abundance of peritoneal mesothelial claudin-2 and arteriolar claudin-3 than in controls. PD patient samples had highest mesothelial and arteriolar claudin-1 and mesothelial claudin-2 abundance, while mesothelial and arteriolar claudin-3 were lowest. Data are presented as median (IQR). One-way ANOVA with Holm-Sidak multiple comparison post-test or Kruskal–Wallis test with Dunn’s multiple comparison post-test were used, accordingly.

**Figure 3:**
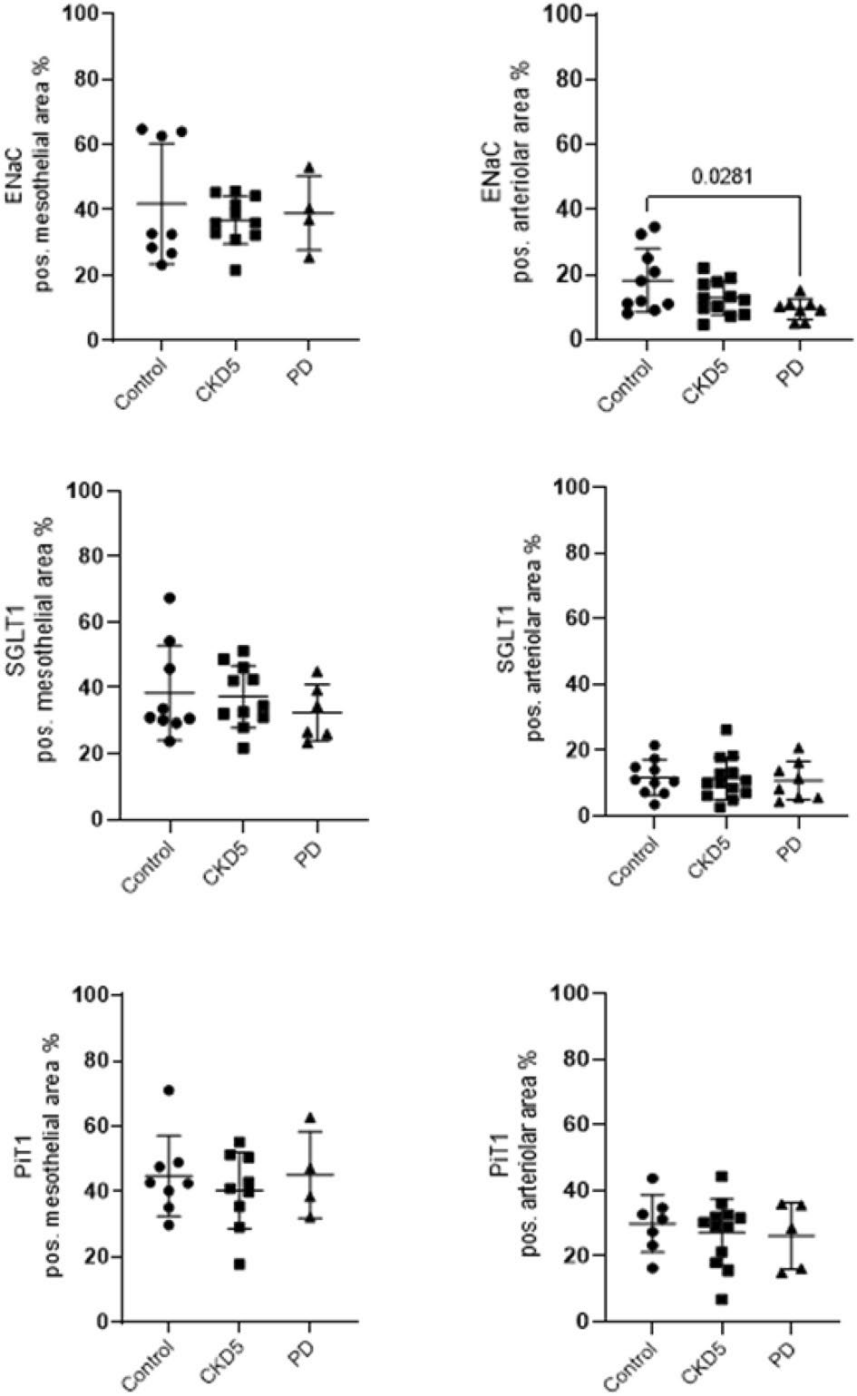
Trancellular sodium channel (ENaC) and sodium and phosphate transporter proteins (SGLT1 and PiT1) in healthy individuals, chronic kidney disease (CKD5) and in peritoneal dialysis (PD). Mesothelial ENaC, mesothelial and arteriolar SGLT1 and PiT1 abundance was unchanged over CKD5 and PD treatment. Arteriolar ENaC abundance was lowest in PD, compared to control values. Data are presented median (IQR). Kruskal–Wallis test with Dunn’s multiple comparison post-test was used.

**Figure 4:**
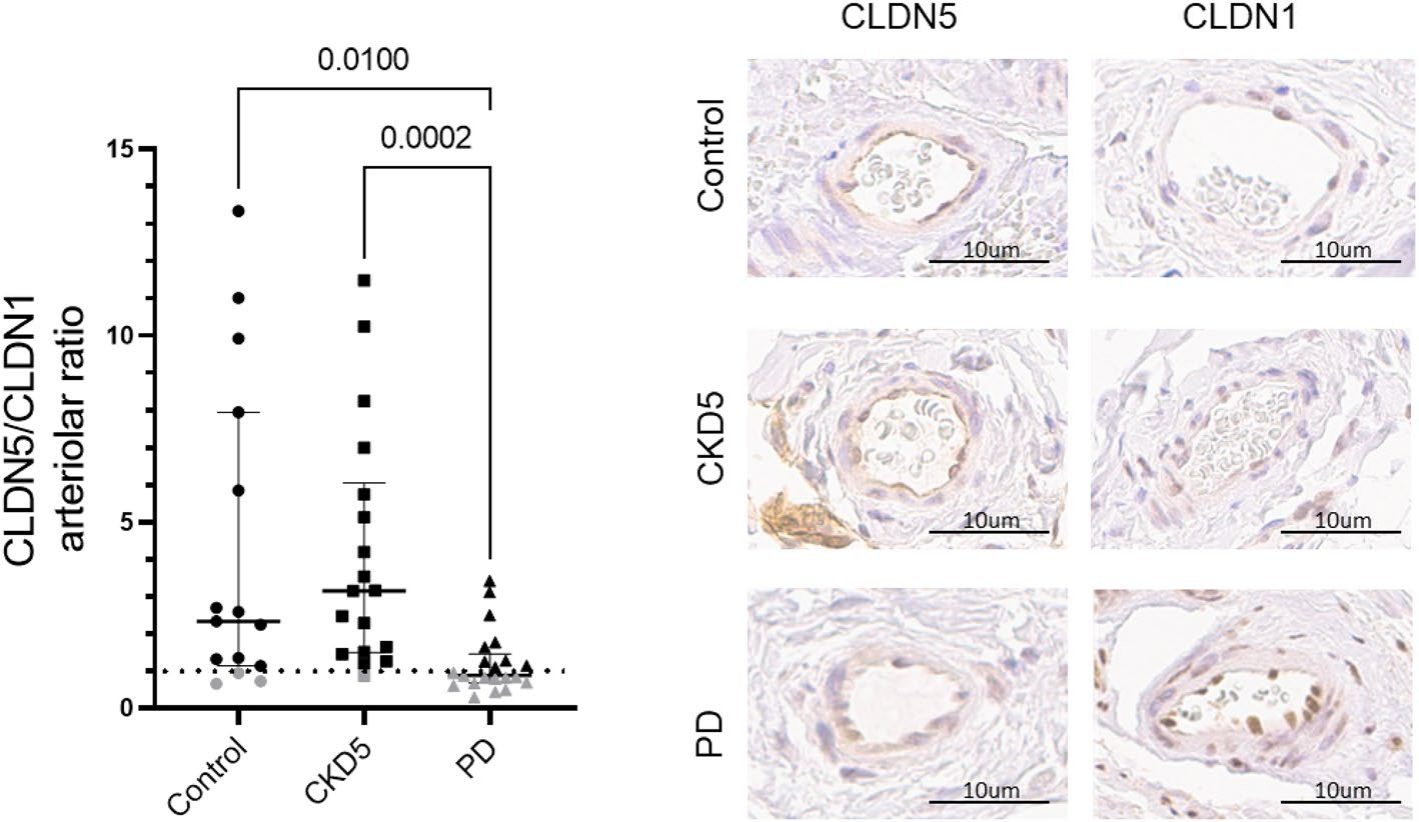
Lower arteriolar CLDN5/CLDN1 abundance ratio in peritoneal dialysis, compared to control and chronic kidney disease (CKD5) indicates an impaired cellular barrier function in PD. Change of the ratio of arteriolar CLDN5 and CLDN1 sealing proteins due to peritoneal dialysis, indicating an impaired sealing capacity of the endothelium and sample stainings from all three investigated groups. Data are presented as median (IQR) and Kruskal–Wallis test with Dunn’s multiple comparison was used. Representative immunostainings of arterioles are given on the right side.

In patients with CKD5, peritoneal mesothelial claudin-2 and arteriolar claudin-3 abundance was higher than in controls. Peritoneum from patients on PD had highest mesothelial and arteriolar claudin-1 and mesothelial claudin-2 abundance, while mesothelial and arteriolar claudin-3 (Fig. 2, Table S1) and arteriolar ENaC were lowest (Fig. 3, Table S1). The arteriolar claudin-5/claudin-1 (CLDN5/CLDN1) ratio indicates impaired cellular barrier sealing function ^30^. Peritoneal arteriolar claudin-5/claudin-1 ratio was 2.3 (IQR 6, 8) in children with normal renal function, 4.14 ± 3.2 in children with CKD5 and 0.88 (0, 78) in children on PD (Kruskal–Wallis *p* = 0,002; Fig. 4). Findings did not differ with history of peritonitis.

**Figure 5:**
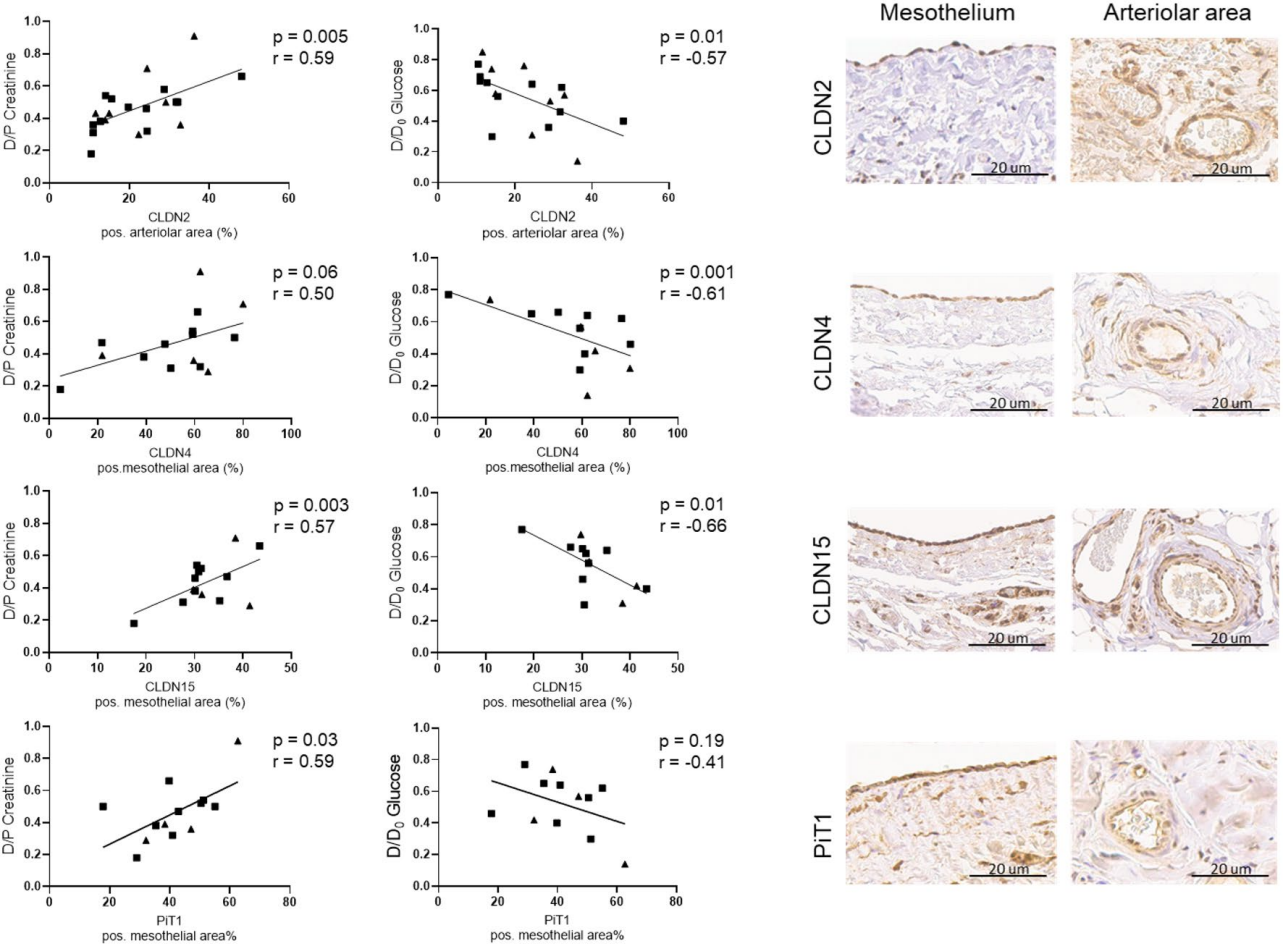
Correlation of arteriolar CLDN2 and mesothelial CLDN4, CLDN15 and PiT1 abundance with D/P creatinine and D/D_0_ glucose obtained from peritoneal equilibration test data in a subcohort of patients at the start of treatment and on chronic peritoneal dialysis. D/P Creatinine and D/D_0_ Glucose significantly correlated with CLDN2 in the arteriolar area, CLDN15 in the mesothelial area and D/D_0_ Glucose with CLDN4 (with D/P Creatinine *p* = 0.06, r = 0.50) in mesothelium and D/P Creatinine with PiT1 (with D/D_0_ Glucose *p* = 0.19, r = -0.41). Representative immunostaining of parietal peritoneum and of arterioles are given.

Dialysate/plasma ratios (D/P) for creatinine and dialysate glucose over initial dialysate glucose concentration ratio (D/D_0_). obtained after 2 h of peritoneal equilibration tests (PET) were 0.46 ± 0.16 and 0.56 ± 0.21. Both correlated with arteriolar claudin-2 and with mesothelial claudin -15 abundance. Mesothelial claudin-4 correlated with D/D_0_ glucose. and mesothelial sodium/phosphate cotransporter PiT1 with D/P creatinine (Fig. 5). When protein quantification was confined to the arteriolar endothelium, claudin-2 correlations with D/D_0_ glucose and D/P creatinine were in the same direction (r = -0.44, *p* = 0.04; r = 0.34, *p* = 0.11). In multivariable analysis including arteriolar claudin-2, submesothelial vessel density (as quantified by CD31 positivity) and age, only arteriolar claudin-2 predicted D/P creatinine and D/D_0_ glucose ratios (*p* = 0.086/0.036, Table 3).

**Table 3:** Multivariable linear regression of D/P creatinine (A) and D/D_0_ glucose (B) in CKD5 and PD patients with peritoneal equilibration test (PET) data (n = 22).

(A)	Multivariable analysis	
	Coeff. (95% CI)	*p*-value
Microvessel density (/ mm^2^)	0.000 (− 0.001, 0.001)	0.413
Age (years)	− 0.007 (− 0.018, 0.004)	0.218
Arteriolar CLDN2 (% pos. area)	0.006 (− 0.001, 0.013)	0.086

(B)	Multivariable analysis	
	Coeff. (95% CI)	*p*-value
Microvessel density (/ mm^2^)	0.000 (− 0.001, 0.001)	0.664
Age (years)	0.004 (− 0.011, 0.018)	0.600
Arteriolar CLDN2 (% pos. area)	− 0.010 (− 0.018, − 0.001)	**0.036**

## Discussion

The peritoneum plays a key role in abdominal homeostasis, in the development of post-inflammatory and post-interventional adhesions, ascites formation and cancer progression^31,32^, all processes in which TJs are essentially involved. Nephrologists take advantage of the semipermeable peritoneum as a natural dialysis membrane for the rising number of patients with end stage kidney disease, but due to PD fluid bioincompatibility progressive peritoneal alterations limit its use. We provide the first in-depth analysis of barrier forming sealing TJ, pore forming TJ exerting paracellular transport, and of key cellular sodium channel and transporters, across age groups, and in patients with CKD and on PD. Since peritoneal solute transport can be measured in patients on PD, these patients provide a unique opportunity for a better understanding of peritoneal TJ and cellular transporter function. The children studied were devoid of life-style and aging related factors and therefore allow for a highly sensitive and specific analysis.

In the healthy peritoneum the nine TJ components and the three transcellular sodium channel and transporters studied are consistently expressed in the mesothelium, in peritoneal microvessels and in the endothelium of peritoneal arterioles, suggesting that mesothelial and endothelial cell monolayers define the peritoneal barrier and transport function. With the exception of mesothelial claudin-4 and arteriolar claudin-1 and -2, findings were consistent across age groups, suggesting that age-dependent differences in peritoneal solute and water transport may primarily be due to differences in peritoneal vascularization^19^.

In children with CKD the peritoneal vessel density was increased, possibly to compensate the concomitant arteriolar lumen obliteration; findings which are both in line with previous studies in larger pediatric cohorts^14^; the molecular mechanisms, however, have not yet been studied. In CKD uremic toxins accumulate, inflammatory and oxidative stress is increased^33,34^. Experimental studies in vitro and in animals suggest uremic toxins, inflammation and oxidative stress induce impairment of TJ expression in the intestine, endothelium, liver, kidney, lung and brain^35–37^. In the peritoneum of children with CKD5 at time of PD catheter insertion, i.e. the most advanced stage of CKD, we only observed an increase in arteriolar sealing claudin-3 and in mesothelial pore forming claudin-2.

In the peritoneum obtained from patients on PD, claudin-1 was more and claudin-3 less abundant in the mesothelium and arterioles than in patients with CKD. Both have sealing functions, and claudin-3 is the only claudin which forms complexes with all other proteins of the claudin family^38,39^. Expression of ENaC, PiT1 and SGLT1 was neither modified by CKD5, nor during PD. The net effect of the TJ regulation on the cellular barrier is uncertain, especially since expression levels do not necessarily reflect TJ function. TJ protein expression, subcellular localization and function vary with tissue- and cell-types^40^. TJ clustering within the cell membrane defines paracellular permeability, and is altered, e.g. by glucose degradation products present in PD fluids^41^.

Peritoneal solute transport and dialytic protein loss increase with time on PD, i.e. peritoneal barrier function is altered with chronic PD^42^. The most striking morphological finding in patients on PD with pH neutral, low GDP PD fluids is the two-fold increase in peritoneal vessel density, demonstrated in a previous study^14^ and reconfirmed now. Peritoneal vessel density independently predicts peritoneal small solute transport^14^. Expression of the pore forming junctions quantified in the arteriolar endothelium was neither altered in CKD nor in patients on PD. Arteriolar expression of claudin-2, a TJ involved in sodium and water transport^43^ correlated with peritoneal creatinine and glucose transport rates, suggesting a major functional involvement of this TJ component in peritoneal solute transport. Similar findings were obtained, when the analysis was confined to the arteriolar endothelium. Expression of mesothelial claudin-4, of which the function depends on the complexes formed with other claudins, including claudin-2, and which can act as a pore- or a selective barrier for sodium transport^44^ also correlated with peritoneal creatinine and glucose transport rates, as did the mesothelial expression of claudin-15, a sodium and water channel^45^. Mesothelial PiT1 correlated with creatinine transport rates.

The specific contribution of the mesothelial cell barrier relative to the endothelial cell barrier for transperitoneal solute transport is still unknown. Our findings suggest a functional impact of the mesothelial TJ in solute transport, and deserve TJ and cell type specific validation in experimental settings. In multivariable analyses including arteriolar TJ abundance, age and vessel density only arteriolar claudin-2, but none of the other TJ, which in univariate analyses were correlated with peritoneal transport, independently predicted the solute transporter status of the peritoneum. The independent prediction of small solute transport by a single claudin, claudin-2, is especially noteworthy in view of the complexity of the TJ system and the heterotypic interactions between different TJ proteins^46^.

The significance of molecular determinants of peritoneal permeability has recently been demonstrated for the peritoneal water selective AQP1 channel, which in mice exerts about 50% of water transfer^47^. PD patients carrying a variant in the AQP1 promoter region resulting in reduced AQP1 function achieve less fluid removal and have an increased mortality, highlighting the important role of single peritoneal transport pathways^23^. They represent druggable targets to increase peritoneal membrane transport function. Pharmacological modulation of AQP1 increased fluid removal in animal models of experimental PD^48^.

Dialytic protein losses increase with time on PD. Our finding of a reduced peritoneal arteriolar CLDN5/CLDN1 ratio due to an increased claudin-1 expression suggests altered vascular sealing, i.e. impairment of the vascular barrier integrity in PD patients, but the role of both claudins and their ratio in peritoneal transport function is yet uncertain. In mice ischemia/reperfusion models, the CLDN5/CLDN1 ratio of the blood/brain barrier inversely correlates with the post-ischemic inflammatory response^30,49,50^. In the peritoneum, we could not demonstrate a correlation of arteriolar CLDN5/CLDN1 with submesothelial CD45 and CD68 cell counts, however, this could be explained with differences in the inflammation pattern induced by PD and in the blood–brain barrier. Dialytic protein loss may reflect generalized endothelial dysfunction and is independently predicted by the D/P creatinine and the appearance rate of IL-6, a marker of local peritoneal inflammation^51^. In a randomized cross-over trial, addition of alanyl-glutamine to the PD fluid, reduced peritoneal protein loss^52^. In mice alanyl-glutamine upregulated peritoneal claudin-5 expression, and in endothelial cells increased claudin-5 and ZO-1 abundance, clustering and transendothelial electrical resistance^53^. These findings suggest a significant role of claudin-5 in peritoneal membrane barrier function, which can be restored by pharmacological means.

Our study has several limitations. The function of the peritoneal paracellular TJ components, of the transcellular sodium channel and of the transporters has only been described in other organs, specific transport function may differ in the human peritoneum and involve other transport associated proteins. We demonstrate associations between peritoneal TJ expression and the small solute transport, i.e. peritoneal creatinine and glucose transport, but no mechanistic links. Peritoneal transport of larger molecules reflecting function of TJ such as tricellulin^54^ and of water has not been studied. Another limitation is the lack of quantifiability of TJ and cellular ion channel and transporters in the peritoneal capillaries, which have been identified as the primary structure for solute and water exchange^55,56^. Single submesothelial endothelial cells cannot precisely be annotated and analyses of the entire submesothelial space includes TJ and cell transporter positive inflammatory cells and erythrocytes, introducing a major bias. In contrast, the mesothelium and arterioles studied were devoid of inflammatory cells, ruling out a respective bias at these sites. In addition, our present findings point to a significant role of the mesothelium cell-layer, and demonstrate distinct alterations in CKD and during PD, with CLDN2 abundance independently predicting transperitoneal small solute transport rates. Our transport studies are based on 2-h PET data, which in children has been shown to provide similar findings as with 4-h PET^57,58^.

## Conclusion

We for the first time comprehensively describe key peritoneal sealing and pore forming TJ that are mediating the paracellular transport, and transcellular transporters and channel across age groups, in CKD and in patients on PD as well as their relation with small molecule transport. Arteriolar claudin-2 independently predicted peritoneal small solute transport rates. Since associations do not inform on mechanistic links, our findings require independent, experimental validation to inform future studies addressing therapeutic interventions in PD and diseases involving the peritoneal transport machinery.

## Materials and methods

### Patient cohort and sampling of the international peritoneal biobank

Peritoneal tissues were collected within the International Peritoneal Biobank (IPPB, registered at www.clinicaltrials.gov—NCT01893710) as described previously^19^. The study was performed according to the Declaration of Helsinki which sets ethical principles regarding human experimentation. The Ethical Committee of the Medical Faculty at the University of Heidelberg and institutional boards from all participating centers approved the study protocol and consent forms. Written informed consent was obtained from the patient’s parents and patients as appropriate. The 46 individuals with normal renal function for analysis of age-related junction and transcellular transporter abundance were analyzed (age 0–75 years), 23 children with CKD5 and 24 children on PD for 12.8 (IQR 7.9, 21.9) months with neutral pH PD fluids with low glucose degradation product content.

Individuals with a body mass index (BMI) of > 35 kg/m^2^ and with chronic inflammatory diseases, and diseases affecting the peritoneum were excluded. Tissues in individuals with normal renal function were collected during abdominal surgeries unrelated to kidney and during living donor kidney transplantation. Tissues from children with CKD5 were obtained at the time of PD catheter insertion, in PD patients at the time of catheter revision and/or exchange, kidney transplantation, hernia/leakage, described in the Supporting Information Methods. PD patients received peritoneal dialysis for median 13 (IQR 8, 22) months before tissue sampling. Dialytic glucose exposure was 115 (74, 152) g/m^2^/day. 29% (n = 7) of patients have experienced peritonitis episodes, but these were all successfully treated and the biopsies were 38 (16, 156) weeks thereafter. Parameters of children with normal kidney function, patients with CKD5 and patients on PD were compared matched for age and body surface are (BSA). Biochemical characteristics are given in Table 1.

In 23 patients (21 on cycling PD), peritoneal equilibration test (PET) was performed to assess peritoneal transport function. PET was performed according to standard guidelines and 2 h D/P creatinine and D/D_0_ glucose were measured^57–59^. The underlying diseases of these 23 patients and their biochemical findings did not differ from the rest of the group.

### Histological studies

Immunohistochemical stainings were performed on formalin-fixed tissue sections according to standard methods and as described previously^14^. All markers were stained by standard immunohistochemistry as described previously (20) with following antibodies: ASMA (Dako Cytomation, Denmark, 1:1000), calretinin (Cell Marque, CA, USA, 1:100), CD31 (1:25), CD45 and CD68 (both 1:100), podoplanin (1:1000) were from Dako Cytomation, Denmark. Claudin-1–5, OCL, TRiC and ENaC were from Thermo Fisher Scientific, MA, USA (all 1:500), ZO-1 (LifeSpan Biosciences, USA, 1:500), SGLT1 (Millipore, USA, 1:2000), PiT1 (SLC20A1) (Thermo Fisher Scientific, MA, USA, 1:500). Secondary antibodies (against the host species of the first antibody were purchased from Thermo Fisher Scientific, MA, USA, 1:300). Immunofluorescence stainings were performed according to standard methods. After dewaxing, heat induced antigen retrieval was performed in microwave. Claudin-5 conjugated with Alexa 488, ZO-1 conjugated with Alexa 555 (Thermo Fisher Scientific, MA, USA, 1:1000) and claudin-2 primary antibody was applied overnight and after washing, secondary Alexa 647 (Thermo Fisher Scientific, MA, USA, 1:1000) antibody added. Cell nuclei were counterstained with DAPI (Thermo Fischer, MA, USA, 1:1000).

Submesothelial thickness was measured at least 5 different sites of CD31 stained scanned sections. Microvessel density was analyzed from CD31 stained tissues and was defined as the number of vessels per unit of analysis area. Podoplanin and CD31 positive vessels were defined as lymphatics. Blood vessel density was calculated from the density of CD31 stained capillaries minus podoplanin positive lymphatics. Diffuse podoplanin staining phenotype was defined as extra-lymphatic (podoplanin positive, but CD31 negative) podoplanin abundance as previously described^60^. Capillary vessel area was defined as the sum of endothelial area plus the lumen area and capillary wall divided by the intimal thickness. The capillary endothelial surface area relative to peritoneal volume was calculated by the endoluminal perimeter of CD31 stained endothelium × section thickness × number of vessels divided by the analyzed peritoneal area × section thickness (µm^2^/µm^3^).

CD45 positive leukocytes, CD68 positive macrophages and ASMA positive cells were quantified per mm^2^ of submesothelial area. Semi-quantitative score was applied for mesothelial coverage (0–6, with 0 = no or isolated cells present only, and 6 representing complete coverage). Mesothelial cells positive for calretinin, present in the submesothelium with phenotypic signs of fibroblasts were defined as EMT cells and quantified per mm^2^ submesothelium. Arteriolar luminal diameter to vessel external diameter (L/V) was quantified on arterioles with a 60–100 μm diameter, average of L/V of 5 to 7 vessels per sample measured was taken as the representative value^14^.

### Digital image analyses

Whole tissues slides were scanned and evaluated using the Aperio® Precision Image Analysis Software as described previously^14,41^. For quantification of junction and transcellular transporting proteins, positive Pixel Count Algorithm (Aperio® Technologies, Inc., Vista, California, USA version 9) was used and regions of interest (ROI) were annotated, excluding surrounding fat tissue and lumen. Intensity ranges were validated for each specific staining, and a threshold set for defining pixel positivity. Protein abundance was calculated as the number of positive pixels divided by total number of pixels, the latter being defined by the ROI area. Tissues for one marker were stained in one run, in case when more than one run was necessary, internal controls were used to normalize staining intensities to account for inter-staining variation. No inflammatory cells were present in the mesothelial cell layer and only arterioles without inflammatory cell infiltration were analyzed.

Confocal microscopy imaging z-stacks of DAPI, Alexa-488, Alexa-555 and Alexa-647 were acquired at × 400 magnification with Leica TCS SP5 (Leica Biosystems, Wetzlar, Germany) confocal microscope. Subsequent co-localization and z-projection with maximal intensity were prepared using open-source FIJI software.

### Statistics

Data are presented either as means (SD) or medians (interquartile range, IQR) based on normality testing by Shapiro–Wilk test. For parametric data t-test was performed for comparisons between two groups, one-way ANOVA for comparisons between three groups (with Holm-Sidak multiple comparison post-test) and for non-parametric data Mann–Whitney U Test and Kruskal–Wallis test (with Dunn’s multiple comparison post-test) were used. χ^2^ or Fisher’s exact test for describing differences in proportions were used. Associations were studied by Pearson and Spearman correlation analysis based on data distribution. In all cases two-sided tests were performed. Multivariable linear regression models were used to test associations of arteriolar claudin-2 abundance with age and microvessel density. GraphPad Prism software (Version 9, La Jolla, CA, USA) and SPSS (Version 25) were used.

### Ethical approval

The study has been approved by all local institutional review boards.

### Informed consent

Patients and parents provided written informed consent, children as appropriate and approved by the local institutional review boards.

### Supplementary Information

Supplementary Information.

## Data Availability

Supporting data is available from the corresponding author upon reasonable request, national and international General Data Protection Regulations apply.
