# Supplementary material for: Human peritoneal tight junction, transporter and channel expression in health and kidney failure, and associated solute transport
Source: Sci Rep. 2023 Oct 13;13:17429. doi: 10.1038/s41598-023-44466-z (PMC10575882; doi:10.1038/s41598-023-44466-z)
Supplement: Supplementary file 1 — Supplementary Information. [file 41598_2023_44466_MOESM1_ESM.pdf]

Supplementary Information for

Human peritoneal tight junction, transporter and channel expression in health and kidney failure, and associated solute transport

Eszter Levai[*1,2,3], Iva Marinovic[*1], Maria Bartosova[*1], Conghui Zhang[1], Betti Schaefer[1], Hanna Jenei[1], Zhiwei Du[1], Dorota Drozdz[4], Günter Klaus[5], Klaus Arbeiter[6], Philipp Romero[7], Constantin Schwab[8], Attila J. Szabo[2,3], Sotirios G. Zarogiannis[#1,9], Claus Peter Schmitt[#1]
*/# contributed equally

[1] Center for Pediatric and Adolescent Medicine, University of Heidelberg, Heidelberg, Germany, 69120
[2] Pediatric Center, MTA Center of Excellence, Semmelweis University; Budapest, Hungary, 1083
[3] ELKH-SE Pediatrics and Nephrology Research Group, Budapest, Hungary, 1083
[4] Jagiellonian University Medical College, Krakow, Poland, 31-530
[5] KfH Pediatric Kidney Center, Marburg, Germany, 35043
[6] Department of Pediatrics and Adolescent Medicine, Medical University Vienna, Austria, 1090
[7] Division of Pediatric Surgery, Department of General, Visceral and Transplantation Surgery, University of Heidelberg, Heidelberg, Germany, 69120
[8] Institute of Pathology, University of Heidelberg, Heidelberg, Germany, 69120
[9] Department of Physiology, Faculty of Medicine, University of Thessaly, Larissa, Greece, 415 00

Short running title: Peritoneal tight junctions, transporter and channel expression

Corresponding author
Claus Peter Schmitt, MD, PhD
Division of Pediatric Nephrology
Center for Pediatric and Adolescent Medicine
Im Neuenheimer Feld 430
69120 Heidelberg, Germany
Phone +49-6221-56-39313
Fax: +49-6221-56-4203
Email: clauspeter.schmitt@med.uni-heidelberg.de

Supplementary Text

Extended Methods

Study population

Peritoneal tissues of 70 children and 23 adult individuals were studied. Patients with a BMI of >35 kg/m2 and with chronic inflammatory diseases, or conditions affecting peritoneal of vascular integrity were excluded.

Underlying diseases in our CKD5 and PD pediatric patients were congenital abnormalities of the kidney and urinary tract (13 and 9 cases), nephronophthisis (4, 4) congenital nephrotic syndrome (3, 3), glomerulopathies (3, 3) autosomal recessive polycystic kidney disease (0, 1) and cystinosis (0, 1), hypoxic renal damage (0, 1).

Occasions of biopsy sampling in control children were exploratory laparoscopy (2 cases), laparoscopic fundoplication (11), laparoscopic cholecystectomy (2), splenectomy (3), pyeloplasty (1) and unknown (1). In patients with CKD5 biopsies were taken at time of Tenckhoff-catheter insertion, in patients on PD at time of catheter revision/exchange (2), kidney transplantation (15) and other occasions (7).

10 out of the 24 low-GDP treated children used a double-chamber PD fluid containing 34 mmol/L of bicarbonate (BicaVera®; Fresenius Medical Care, Bad Homburg, Germany), 7 children a double-chamber PD fluid containing 35 mmol/l of lactate (Balance®, Fresenius Medical Care, Bad Homburg, Germany) and 7 children a double chamber fluid containing 25 mmol/L bicarbonate and 15 mmol/L lactate (Physioneal®; Baxter Healthcare Corporation, Deerfield, IL, USA). The PD

fluids were similar in pH (7-7.4), electrolyte composition, and osmolarity, and only slightly differed in GDP content.

Underlying diseases of the cohort were not affecting the transport characteristics. PET was performed according to standard guidelines and 2h D/P creatinine and D/D_0 glucose were measured.

Two adult individuals from the age-dependency control group were excluded due to inadequate tissue quality.

Supplementary Figure

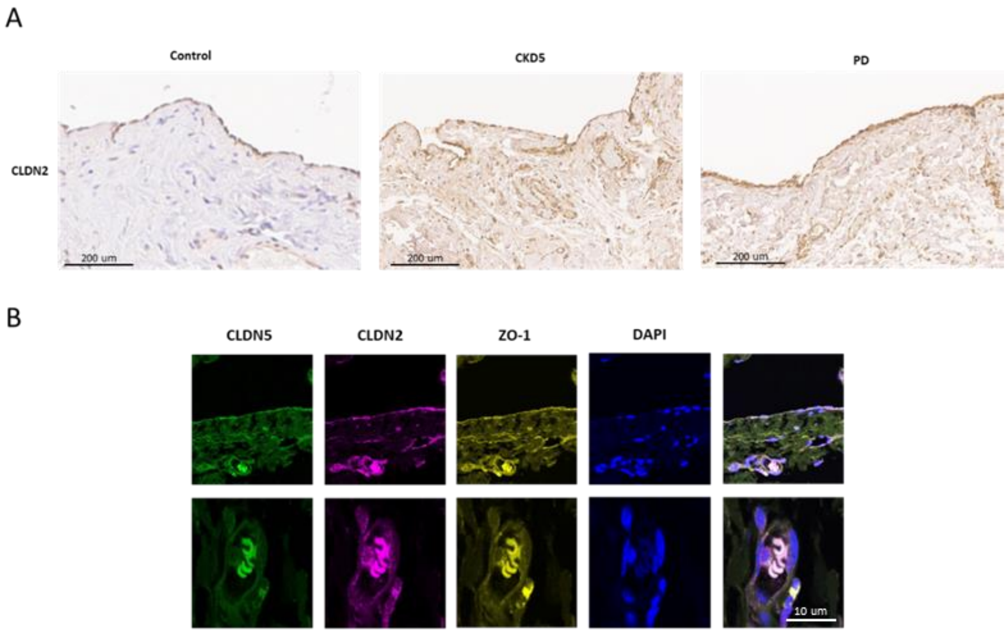

Figure S1. Claudin-2 in the mesothelium and submesothelium (A) with inflammatory cell infiltration and co-staining of CLDN5, CLDN2, ZO-1 and DAPI of the mesothelium (B - upper row) an arteriole and a leukocyte (B-lower row).
To exclude the analytic bias by the degree of inflammatory cell infiltration, we limited our studies to the mesothelial cell monolayer (**A, B - upper row**), where no inflammatory cells were present, and the arteriolar area (**B – lower row**). Arterioles with presence of inflammatory cells (CD68, CD45) were excluded from the analysis.
Confocal microscopy imaging z-stacks of DAPI, Alexa-488 (CLDN5), Alexa-555 (ZO-1) and Alexa-647 (CLDN2) were acquired with Leica TCS SP5 (Leica Biosystems) confocal microscope. (**B**)

Supplementary Tables

Table S1. Tight junction and transcellular transporter protein-abundance in mesothelial and arteriolar area (% positivity relative to total stained area)

	Controls (n=23)	CKD5 (n=23)	PD (n=24)	p value*
		**Mesothelial area**		
		Tight junction proteins		
**CLDN2**	15.8 ± 8.1	56.0 ± 7.2	64.9 ± 9.6	**p<0.0001**
**CLDN4**	52.8 ± 7.9	53.8 ± 15.4	54.6 ± 14.9	0.80
**CLDN15**	25.9 (16.2, 34.8)	30.2 (17.6, 32.3)	17.0 (13.8, 31.5)	0.52
**CLDN1**	12.7 ± 8.1	22.3 ± 12.7	32.8 ± 15.5	**0.001**
**CLDN3**	46.4 ± 17.3	52.3 ± 23.3	31.0 ± 7.2	**0.02**
**CLDN5**	35.8 (20.2, 39.4)	46.7 (33.7, 57.5)	34.8 (16.5, 45.1)	0.1
**ZO-1**	32.8 (13.4, 59.5)	43.8 (29.5, 56.4)	48.2 (44.6, 52.3)	0.07
**OCL**	78.6 ± 8.4	77.3 ± 14.6	74.3 ± 8.8	0.52
**TriC**	29.0 ± 16.0	30.8 ± 13.3	30.3 ± 11.0	0.93
		Transcellular channel and transporters		
**ENaC**	32.7 (27.2, 63.6)	36 (32.3, 44.3)	38.8 (28.4, 50)	0.97
**SGLT1**	31 (29.8, 50)	34.4 (31.1, 46,1)	30.5 (25.4, 40.6)	0.53
**PiT1**	42.6 (36.4, 48.6)	40.9 (32.2, 50.8)	42.8 (33.7, 58.8)	0.95
		**Arteriolar area**		
		Tight junction proteins		
**CLDN2**	16.6 ± 8.8	23.6 ± 11.5	21.9 ± 11.2	0.08
**CLDN4**	37.6 ± 10.7	32.3 ± 12.1	30.5 ± 14.6	0.19
**CLDN15**	13.7 ± 6.1	16.9 ± 6.6	12.3 ± 6.9	0.19
**CLDN1**	26.8 ± 21.4	16.8 ± 13.3	46.1 ± 24.4	**p<0.0001**
**CLDN3**	29.6 ± 18.1	47.8 ± 18.4	19.3 ± 10.2	**0.002**
**CLDN5**	38.9 ± 13.5	47.0 ± 11.6	48.3 ± 18.5	0.11
**ZO-1**	21.2 ± 12.8	27.6 ± 10.7	24.2 ± 7.9	0.15
**OCL**	71.5 (55.6, 73.3)	69.1 (41.5, 74.9)	60.5 (44.0, 70.1)	0.26
**TriC**	44.8 ± 11.0	44.1 ± 18.9	44.9 ± 20.8	0.99
		Transcellular channel and transporters		
**ENaC**	18.3 ± 9.8	12.8 ± 5.3	9.5 ± 3.2	**0.01**
**SGLT1**	11.8 ± 5.4	11.5 ± 6.4	10.8 ± 5.9	0.94
**PiT1**	29.9 ± 8.7	27.2 ± 10.1	26.2 ± 10.1	0.78

*Data are mean ± SD or median and interquartile range. *ANOVA or Kruskal Wallis test as appropriate.*

Table S2. Tight junction protein and transcellular channel and transporter abundance across age-groups in positive area of stained mesothelial and arteriolar area (%) in children and adults with normal kidney function.

	**0-2 y (n=8)**	**3-11 y (n=10)**	**12-17 y (n=5)**	**18-59 y (n=14)**	**60-75 y (n=9)**	**p-value***
			**Mesothelial area**			
			Tight junction proteins			
**CLDN2**	16 (10, 18.9)	14.9 (10.3, 23.3)	13 (6.6, 32.2)	18.5 (9, 34.1)	16.1 (10.1, 24.2)	0.9
**CLDN4**	53.9 (46.5, 56.8)	51.6 (44, 58.2)	58.7 (52.8, 64.3)	22.5 (15.3, 51.2)	39.7 (34.6, 61)	**0.02**
**CLDN15**	22 (14.9, 36.5)	22 (11.7, 38.2)	29.6 (20.2, 34.3)	n/a	n/a	0.81
**CLDN1**	12 (4.8, 21.5)	16.6 (3.2, 46.6)	12.2 (8.2, 15.1)	18.1 (7.1, 21.3)	25.8 (24.5, 27.1)	0.46
**CLDN3**	46.7 (29, 50)	66.6 (34, 72.8)	39.4 (28.9, 46.2)	54.5 (53.5, 55.6)	45.3 (43.2, 47.3)	0.23
**CLDN5**	38.3 (16.7, 45.9)	35.7 (21, 42.4)	29.5 (17.9, 35.9)	26.8 (17.9, 45.8)	36.2 (33.6, 45.7)	0.71
**ZO-1**	25 (16.4, 51.2)	19.2 (4.1, 74.3)	57.8 (20.9, 86,6)	71.2 (38.1, 148.4)	33.4 (8, 63.4)	0.29
**OCL**	75.8 (62.1, 81)	77.8 (75.6, 87.2)	73.3 (48.4, 84.7)	75.1 (61.8, 82.6)	66.4 (55.5, 79.8)	0.39
**TriC**	30.8 (13.6, 48.3)	20 (12, 41.9)	31 (15.1, 51.5)	24.3 (23.7, 55.6)	n/a	0.72
			Transcellular channel and transporters			
**ENaC**	23.1 (1.4, 64.7)	48.3 (32.6, 64.0)	32.8 (28.5, 62.6)	n/a	n/a	0.76
**SGLT1**	30.2 (1.0, 54.2)	45.8 (33.6, 67.4)	30.6 (29.3, 31.0)	n/a	n/a	0.23
**PiT1**	22.9 (3.1, 42.7)	40.2 (29.7, 48.9)	47.6 (42.5, 71.0)	n/a	n/a	0.43
			**Arteriolar area**			
			Tight junction proteins			
**CLDN2**	16 (8.6, 28)	16.5 (7.4, 20.3)	24 (14.5, 51.9)	11.9 (7.9, 23.9)	3.2 (2.2, 9.1)	**0.005**
**CLDN4**	34.4 (25.2, 47.1)	34.5 (29.6, 44.5)	40.8 (34.3, 48.2)	26.7 (21.7, 36.2)	33 (30.2, 42.7)	0.24
**CLDN15**	12 (8, 16.1)	13.6 (12.8, 19.6)	11.2 (8.9, 12.18)	n/a	n/a	0.2
**CLDN1**	4.4 (2.7, 20.6)	36.7 (22.9, 57.4)	30.6 (10, 32.1)	5 (2.1, 7.6)	26.0 (23.5, 37.7)	**0.005**
**CLDN3**	41.7 (13, 48.3)	25.4 (12.1, 55.8)	23.2 (11.6, 37.8)	26.1 (5.9, 41.9)	31.8 (25.5, 39.7)	0.89
**CLDN5**	35.2 (31.5, 48)	34.3 (28.4, 45.1)	39.9 (34, 66.9)	41.6 (23.2, 56.6)	43.9 (38.3, 49.6)	0.69
**ZO-1**	11.2 (3.4, 26.3)	21.6 (17.5, 31.4)	30.4 (15.2, 45.2)	31.1 (21.9, 40.6)	20.4 (1.6, 35.1)	0.12
**OCL**	58.2 (39.6, 66.9)	73 (71.3, 77)	72.6 (45.1, 75.5)	61.4 (44.2, 76.8)	62.3 (58, 63.4)	0.08
**TriC**	46.3 (36.2, 53.7)	44.8 (33.4, 54.8)	49.2 (38.7, 57.4)	42.1 (40.5, 47.1)	n/a	0.84
			Transcellular channel and transporters			
**ENaC**	11.2 (8.1, 34.7)	16.4 (9.8, 24.1)	18.1 (11.0, 32.5)	n/a	n/a	0.94
**SGLT1**	10.6 (3.6, 21.6)	8.7 (7.0, 15.7)	14.1 (11.1, 14.9)	n/a	n/a	0.6
**PiT1**	23.3 (23.3, 23.3)	32.8 (27.4, 34.7)	31.3 (16.4, 43.7)	n/a	n/a	0.74

*Data are mean ± SD or median and interquartile range. *ANOVA or Kruskal Wallis test as appropriate. n/a – Not available.*
